# Facts and Analogies Designed to Show That Hydrocyanic Acid Is Probably the Cause of Autumnal Fevers

**Published:** 1829-02

**Authors:** G. S. B. Hempstead

**Affiliations:** Portsmouth, Ohio


Art. II.—*Facts and Analogies designed to show that Hydrocyanic Acid is probably the cause of Autumnal Fevers.* By G. S. B. Hempstead, M. C. of Portsmouth, Ohio.

To *ascertain* and *identify* the causes of epidemic diseases, is certainly a desideratum in our profession. That *Malaria*, or the exhalations from decaying vegetable and animal matter are the cause of our autumnal fevers, by the unanimity of the profession, seems fully established. It is equally certain, that the Dysenteries, Choleras and Diarrhœas of the summer months, owe their origin to the same cause, differently modified. The gases, which result from the process of decomposition, have a specific action on the system; and, uniformly, under similar circumstances, will produce similar effects; whether applied to the stomach or surface, and this through the medium of the nervous system. In proof of which, we will only observe, if an astringent be applied to the stomach, or any part possessing sensibiltiy, by a continuation of sensation its specific effect is excited in a distant part; and because we see its effect in a *diseased* distant part, we say one sympathises with the other; when, in fact, the whole nervous system is, to a certain degree, affected in a similar manner. A blister, by its specific action on the nerves of the part to which it is applied, produces a corresponding effect on the sensibility of the whole system; and thereby relieves disease in a diseased distant part; so of opium, assafœtida, tonics, &c. Cold water applied to one extreme of the body will restrain hemorrhage from the other: This is not from sympathy, for if applied to any other part of the body equally sensible, it would produce a similar effect; nor by revulsion, for it lessens the calibre of the vessels and would force more blood on the distant part; but by a continuation of sensation throughout the whole nervous system, every blood vessel is lessened in its caliber. Hence miasma applied to the nose, fauces, stomach, lungs, and even the surface of the body, is capable of producing many of the phenomena of fever. This gas being stimulant will, after a longer or shorter time, produce indirect debility; consequently from a long continued application of it, we become accustomed to its action, and require a larger dose to produce its specific effect. Besides, from the long continued heat of our climate, our bodies become relaxed, and a state of indirect debility is not so readily induced. Hence natives of low latitudes and marshy countries, are less liable to autumnal diseases, than those from a pure and tonic atmosphere, whose fibres are tense and circulation active. The latter are more readily thrown into a state of indirect debility, a torpor succeeds, and the usual consequences which arise from suspended secretions and excretions must be the result. Our professional brethren, after having ascertained the fact, that the exhalations from decaying vegetable and animal matter, are the principal, or only cause. of autumnal disease, have tacitly agreed to suspend their investigations. Having ascertained that heat and moisture are necessary agents, their labors have ceased, without calling to their aid the assistance which chemistry within a few years has furnished. In fact, we know but little more of the causes of autumnal diseases, than that they exist from the effects which they have on our bodies. The object of these remarks is to reduce, *if possible*, these causes to a tangible form, to give them 'a local habitation and a name.' If we should not succeed in convincing the profession of the *nature of marsh miasmata*, our object will not be lost if we can direct the attention of medical men to an investigation of this interesting and important subject. Chemistry demonstrates clearly to us the constituents of vegetable and animal matters; but in the laboratory we must exclude one agent which is necessarily present in the production of malaria; this agent is atmospheric air, hence it has been imposible to produce the same results from the retort as are produced in the open air. Analysis shows, that from the decomposition of vegetable matter, we obtain hydrogen, carbon and azote, in certain definite proportions; which, when united agreeably to the rule of chemical equivalents, must produce *hydro-cyanic acid*. It may be said, that in the decomposition of vegetable substances, we find the azote in too small a proportion to form the above acid, but this deficiency we supply from the atmosphere by the aid of chemical affinity. The following remarks, from a very celebrated writer, will elucidate our views on this subject. "It has been ascertained, that a clayey soil, when moistened, will attract the oxygen of the air and leave its azotic part not sufficiently guarded, to support life, and it is found, that districts become unhealthy chiefly when the earth begins to appear, in consequence of a diminution of the water. It is singular that Linnæus, with a view to prove the cause of intermittents to be an argillaceous earth, has traced very minutely the prevalence of intermittents in clayey countries; a circumstance which may be explained from the views just assigned." The facts in the above paragraph, we are willing to admit; but we cannot admit, that after abstracting a portion of oxygen from the atmosphere the compound is still atmospheric air, for we believe that the azote is already at the lowest state of oxidation. We would rather explain it thus, that in consequence of the abstraction of oxygen, a portion of azote is left uncombined, and, therefore, ready to unite with any substance with which it may come in contact, for which it has an affinity. It meets with the carbon, hydrogen and a small portion of azote evolved from the vegetable, these combining chemically, in their definite proportions, and there is nothing to forbid it, must produce the *hydro-cyanic acid;* for the carbon and hydrogen of the vegetable, with the azote obtained as above, are very nearly in the definite proportions, to constitute this compound. That this acid is produced by the decomposition of animal matter, we have the testimony of the ablest chemists of our day. Hence diseases may be produced by putrid animal substances, without any decomposition of the atmosphere by moisture; because animal matter contains a sufficiency of azote, without drawing it from a foreign source. "If animal substances be exposed to a high temperature, azote and ammonia, or hydrogen and carbon are produced;" hence the hotest seasons are not the most unhealthy, for the azote is separated, and the ammonia formed, neither of which is deleterious. This acid boils at the temperature of 79° of Fahrenheit, and is highly volatile; hence that season which supports a temperature between 70° and 85°, *cæteris paribus*, is most productive of disease; for experience teaches us, that those seasons which have supported a temperature of from 85° to 95° are comparatively healthy. After a diminution of temperature, the process of decomposition is checked, gas is no longer evolved, and being specifically lighter than atmospheric air, its effects are no longer visible. We are told that this acid is instantly decomposed by contact with chlorine gas. Is not this a strong argument in favour of this identity, from the known efficacy of muriatic fumigations in infectious diseases? Having attempted, by chemical facts, to prove the identity of *marsh miasma* and *hydro-cyanic acid*, we will now compare the effects of each, on the human system, and by analogy endeavour to prove that they are identically the same. The effects of miasmata are, first to stimulate, and then to depress the powers of life; producing prostration of strength, lowness of spirits; unwillingness to move; pain in the head; vertigo; horripilation; chills and tremors; catharsis; emesis; ptyalism; accelerated pulse; loss of sensibility; urtication in those subject to it; convulsions, and an increased secretion from the liver. On adults, it has a more sensible effect than on children. Phthisical and asthmatic patients experience relief from their sufferings, and enjoy a good degree of health and spirits, and are sometimes blessed with a removal of their complaints, by exposure to an atmosphere impregnated with this gas. It is not unfrequently the case, during the prevalence of a severe epidemic, that persons are suddenly deprived of all power of locomotion, all the functions of the system, except the circulation and respiration, cease for a short time; in a few minutes it passes off and they are able to resume their avocations. This has happened frequently during the prevalence of the yellow fever, and in a few instances within our own observation; resembling the effects which are produced by hydro-cyanic acid, when applied to different animals. It is now clearly ascertained, that hydro-cyanic acid may be obtained, in the laboratory, from a variety of vegetables; that some possess it already formed in the woody fibre, and in the seeds, and when thus obtained, may be absorbed by water, and given as a medicine. Those, who have used it most frequently, assert that at first it stimulates, and then distroys sensibility and muscular power, producing unwillingness to move, pain in the head, vertigo, horripilation, cold extremities, catharsis, emesis, ptyalism, accelerated pulse, convulsions, urticaria in those subject to it, and an increased secretion from the liver. On phthisical and ashmatic patients it has a most happy effect, if not to cure, at least greatly to relieve the distress and pain. It has a greater effect on full grown animals, than on those not yet arrived at maturity. We are informed by Dr. Chapman, that he found spirit. terrebin. the most effectual remedy in the bilious vomiting, which occurs in yellow fever; and Orfila declares it to be the only known antidote to the poison of hydro-cyanic acid. Dr. Murray has lately ascertained, that ammonia and acetic acid, in combination, form a valuable antidote to this poison. The value of this mixture, in bilious vomiting and autumnal fever is well known to the profession, Some experiments have been made on this subject which confirm the above facts, but as they were insulated, we shall withhold them until an opportunity shall offer to test them further. Thus far we have attempted, chemically and analogically, to prove the identity of these two articles, and if we have not convinced, we hope, at least, to have so far made proselytes of the profession, as to induce a candid examination of the subject.

